# Accelerated hyperfractionation plus temozolomide in glioblastoma

**DOI:** 10.1186/s13014-016-0645-3

**Published:** 2016-05-21

**Authors:** David Kaul, Julian Florange, Harun Badakhshi, Arne Grün, Pirus Ghadjar, Sebastian Exner, Volker Budach

**Affiliations:** Klinik für Radioonkologie und Strahlentherapie, Charité Universitätsmedizin Berlin, Campus Virchow-Klinikum, Augustenburger Platz 1, 13353 Berlin, Germany

## Abstract

**Introduction:**

Hyperfractionated (HFRT) or accelerated hyperfractionated radiotherapy (AHFRT) have been discussed as a potential treatment for glioblastoma based on a hypothesized reduction of late radiation injury and prevention of repopulation. HFRT and AHFRT have been examined extensively in the pre-Temozolomide era with inconclusive results. In this study we examined the role of accelerated hyperfractionation in the Temozolomide era.

**Materials and methods:**

Sixty-four patients who underwent AHFRT (62 of which received Temozolomide) were compared to 67 patients who underwent normofractionated radiotherapy (NFRT) (64 of which received TMZ) between 02/2009 and 10/2014. Follow-up data were analyzed until 01/2015.

**Results:**

Median progression-free survival (PFS) was 6 months for the entire cohort. For patients treated with NFRT median PFS was 7 months, for patients treated with AHFRT median PFS was 6 months. Median overall survival (OS) was 13 months for all patients. For patients treated with NFRT median OS was 15 months, for patients treated with AHFRT median OS was 10 months. The fractionation regimen was not a predictor of PFS or OS in univariable- or multivariable analysis. There was no difference in acute toxicity profiles between the two treatment groups.

**Conclusions:**

Univariable and multivariable analysis did not show significant differences between NFRT and AHFRT fractionation regimens in terms of PFS or OS. The benefits are immanent: the regimen does significantly shorten hospitalization time in a patient collective with highly impaired life expectancy. We propose that the role of AHFRT + TMZ should be further examined in future prospective trials.

## Introduction

Gliomas are the most common primary tumors of the central nervous system (CNS) in adults representing about one third of central nervous system tumors and 81 % of all malignant CNS tumors reported in the United States [1]. The most common and most malignant type of glioma is glioblastoma (GBM), with a median overall survival (OS) rate of 15 months after surgical resection followed by adjuvant radiotherapy (RT) and Temozolomide (TMZ) chemotherapy. The prevalence of GBM is highest in patients aged 50 years or older and is likely to increase with the ongoing demographic shift toward older ages [2].

Well-known postitive prognostic factors for OS in GBM patients are young age at diagnosis, high Karnofsky performance score (KPS), great extent of neurosurgical resection, O-6-methylguanine-DNA methyltransferase- gene (MGMT) methylation as well as isocitrate dehydrogenase (IDH) 1-mutational status [3–5]. Current standard of care for newly diagnosed GBM comprises maximal safe resection, adjuvant radiotherapy with (RT) with concurrent TMZ and post-RT TMZ chemotherapy [6, 7]. Fractionated RT to the tumor bed in 30 fractions of 2 Gy in single doses of 2 Gy to a total accumulated dose of 60 Gy delivered over the course of 6 weeks has been widely accepted as the standard fractionation regimen, balancing effectiveness with radiation toxicity. Recently some authors have suggested hypofractionated regimens for the elderly and frail patient population [8, 9] other authors have evaluated the role of hypofractionation plus TMZ [10].

Other authors have examined the potential role of hyperfractionated- (HFRT) and accelerated hyperfractionated radiotherapy (AHFRT) as well as the role of protons in GBM [11]. The use of HFRT and AHFRT is based on a hypothesized reduction of late radiation injury and prevention of tumor repopulation in treatment intervals [12].

Despite plausible rationales, various trials have failed to prove the superiority of dose-escalated HFRT and AHFRT in the pre-TMZ era [13].

In 1994, the European Organization for the Research and Treatment of Cancer (EORTC) reported an AHFRT dose escalation trial using doses of 42–60 Gy in 2 Gy fractions three times daily, which failed to show differences in survival in all groups. No additional chemotherapy was used [13]. In 1999 Lutterbach et. al. showed survival rates for 1.5 Gy thrice daily to 54 Gy comparable to conventional RT, again no chemotherapy was used [14]. In 2001 Prados et. al. showed data for AHFRT with or without difluromethylornithine (DFMO) vs. conventional irradiation with or without DFMO with no OS benefit for the experimental groups [15].

The RTOG 83–02 study tested HFRT (2 × 1.2 Gy to doses of 64.8, 72, 76.8, or 81.6 Gy) vs. AHFRT (2 ×1.6 Gy to doses of 48 or 54.4 Gy), all groups received concurrent bis-chloroethyl (BCNU). Contrary to the other aforementioned studies HFRT patients who had received higher doses of 76.8 and 81.6 Gy showed superior survival compared to the AHFRT groups [16].

In summary, the data on HFRT and AHFRT mainly stem from the pre-TMZ era and are not fully conclusive. We therefore want to present experience from our institution on the treatment of patients with newly diagnosed GBM with AHFRT of 2 × 1.6 Gy to 59,2 Gy and concurrent and sequential Temozolomide following the Stupp regimen. Apart from a potential reduction of tumor repopulation as well as a hypothesized reduced late toxicity rate, the regimen does significantly shorten hospitalization time in a group of patients with highly impaired life expectancy.

## Materials and methods

### Treatment decisions, patient selection and dose regimens

Starting from 01/2009 patients with resected GBM with organs-at-risk (OAR) in close proximity to the resection cavity were offered adjuvant radio-chemotherapy (RCTx) with single doses of 1.6 Gy twice daily to a total dose of 59.2 Gy (19 days schedule) as an alternative to a conventional fractionation with single doses of 2 Gy up to 60 Gy (30 days schedule, NFRT). Of 131 patients 126 received continuous daily TMZ (75 mg per square meter of body-surface area per day, 7 days per week from the first to the last day of radiotherapy), followed by six cycles of adjuvant TMZ (150 mg per square meter for 5 days during each 28-day cycle).

In this study we carried out a retrospective analysis of 64 patients who underwent AHFRT plus TMZ and compared them with 67 patients who underwent NFRT plus TMZ between 02/2009 and 10/2014. Follow-up data were analyzed until 01/2015.

In our institution treatment decisions are based on the votes of an interdisciplinary tumor board. Usually all patients <70 years with a KPS >50 % are offered adjuvant AHFRT + TMZ or NFRT + TMZ. AHFRT + TMZ is offered when OARs such as the optic nerves, chiasm or brainstem would be touched by the CTV and covered by the PTV, and in case that the patient is willing and fit enough to undergo treatment twice daily.

Patients ≥70 yeas of age either receive hypofractionated radiotherapy or TMZ only (depending on MGMT-status).

### Stratification, variables and follow-up

Patients were stratified according to fractionation scheme, age, gender, KPS, extent of surgery (biopsy, partial-, gross total resection), MGMT-status, tumor localization (frontal, parietal, temporal, occipital, central) and planning target volume (PTV). Follow-up examinations, including MRI as well as clinical and neurologic examinations were performed at 6–8 week intervals after radiotherapy.

### Treatment planning

Target delineation in GBM varies substantially between different institutions and several consensus statements are available. However, an ESTRO-ACROP guideline is available since January 2016 [17]. Adjuvant RCTx was initiated within 4 weeks after surgical resection or stereotactic biopsy. Contrast agent enhanced computed tomography in a thermoplastic mask as well as gadolinium enhanced magnetic resonance imaging (MRI) was performed before RT planning.

Target volumes were based on preoperative and postoperative MRI. The gross tumor volume (GTV) was defined as the summation of the postoperative surgical cavity with or without residual tumor lesion(s) as well as tumor extension on the preoperative T1-weighted gadolinium-enhanced imaging. The diffusion-weighted imaging (DWI) images were also used in the estimation of GTV. The extent of peritumoral edema was not routinely included in the clinical target volume (CTV), however, an all-round GTV margin of 2 cm was mandatory. For the planning target volume (PTV) an additional 0.5 cm margin was added. Intensity-modulated radiation therapy (IMRT) was applied using a 6-MV linear accelerator with multileaf collimators. Until 2012 treatment was performed using step-and-shoot intensity-modulated radiation therapy (IMRT), starting in early 2012 all patients were treated using volumetric arc therapy (VMAT).

### Toxicity

Higher grade acute toxicity (≥3°) was analyzed for 90 days post treatment according to CTCAE 4.0.

### Formulas and statistics

Overall survival (OS) and progression-free survival (PFS) were calculated from the first day of irradiation using Kaplan-Meier analysis and the log-rank test. Progression was defined retrospectively by clinical note assessments that included integration of imaging and clinical status. Subgroups were compared using univariable analysis and the Cox proportional hazard model for multivariable analysis. A p-value of less than 0.05 was considered statistically significant. A *p*-value of less than 0.1 was considered a trend. All variables from the univariable analysis were included in multivariable analysis. All statistical analyses were performed using IBM SPSS Statistics 19 (New York, USA).

## Results

### Patient characteristics

Patient characteristics are shown in Table 1. One hundred thirty-one patients treated for GBM were identified in our retrospective analysis. Sixty-seven were treated with NFRT and 64 patients were treated using AHFRT.

**Table 1:** Patient characteristics of the 131 GBM patients analyzed

		Overall Collective		NFRT		AHFRT		*p*-value
		(*n* = 131)		(*n* = 67)		(*n* = 64)		
Median Age (min/max) [y]		61	12/80	63	43/78	59	12/80	*p* < 0.001 (*)
Mean PTV ± sd [ccm]		355	±142	339	±141.4	373	±141.8	*p* = 0.17
		n	%	n	%	n	%	
Gender	m	88	67.2 %	46	68.7 %	42	65.6 %	*p* = 0.85
	f	43	32.8 %	21	31.3 %	22	34.4 %	
Localization	Frontal	42	32.1 %	16	23.9 %	26	40.6 %	*p* = 0.38
	Parietal	31	23.7 %	17	25.4 %	14	21.9 %	
	Temporal	38	29.0 %	22	32.8 %	16	25.0 %	
	Occipital	9	6.9 %	4	6.0 %	5	7.8 %	
	Central	9	6.9 %	6	9.0 %	3	4.7 %	
	n/a	2	1.5 %	2	3.0 %	0	0.0 %	
MGMT-status	unmethylated	63	48.1 %	32	47.8 %	31	48.4 %	*p* = 0.66
	methylated	43	32.8 %	23	34.3 %	20	31.3 %	
	n/a	25	19.1 %	12	17.9 %	13	20.3 %	
Extent of surgery	Biopsy	16	12.2 %	6	9.0 %	10	15.6 %	*p* = 0.38
	Partial resection	57	43.5 %	28	41.8 %	29	45.3 %	
	Gross tumor resection	51	38.9 %	29	43.3 %	22	34.4 %	
	n/a	7	5.3 %	4	6.0 %	3	4.7 %	
KPS	50 %	7	5.3 %	4	6 %	3	4.7 %	*p* = 0.3
	60 %	49	37.4 %	27	40 %	22	34.4 %	
	70 %	47	35.9 %	24	36 %	23	35.9 %	
	80 %	28	21.4 %	12	18 %	16	25.0 %	
Temozolomide	yes	126	96.2 %	65	97.0 %	61	95.3 %	*p* = 0.68
	no	5	3.8 %	2	3.0 %	3	4.7 %	
Salvage treatment	Re-irradiation	20	15.3 %	12	17.9 %	8	12.5 %	
	Chemotherapy (tmz)	45	34.4 %	24	35.8 %	21	32.8 %	
	Chemotherapy (other)	6	4.6 %	3	4.5 %	3	4.7 %	
	Bevacizumab	11	8.4 %	5	7.5 %	6	9.4 %	
	Imatinib	1	0.8 %	0	0.0 %	1	1.6 %	
	Dendritic cell vaccination	1	0.8 %	1	1.5 %	0	0.0 %	

*NFRT* normofractionated radiotherapy, *AHFRT* accelerated hyperfractionated radiotherapy, *PTV* planning target volume, *n/a* not applicable, *MGMT* O-6-methylguanine-DNA methyltransferase, *KPS* Karnofsky performance status, *tmz* temozolomide

The two groups were well matched in terms of gender, PTV, tumor localization, MGMT-status, extent of surgery, KPS and TMZ treatment and salvage treatment. Median age in the AHFRT group was lower than in the NFRT group (*p* < 0.001).

### Progression-free survival

**Fig. 1:**
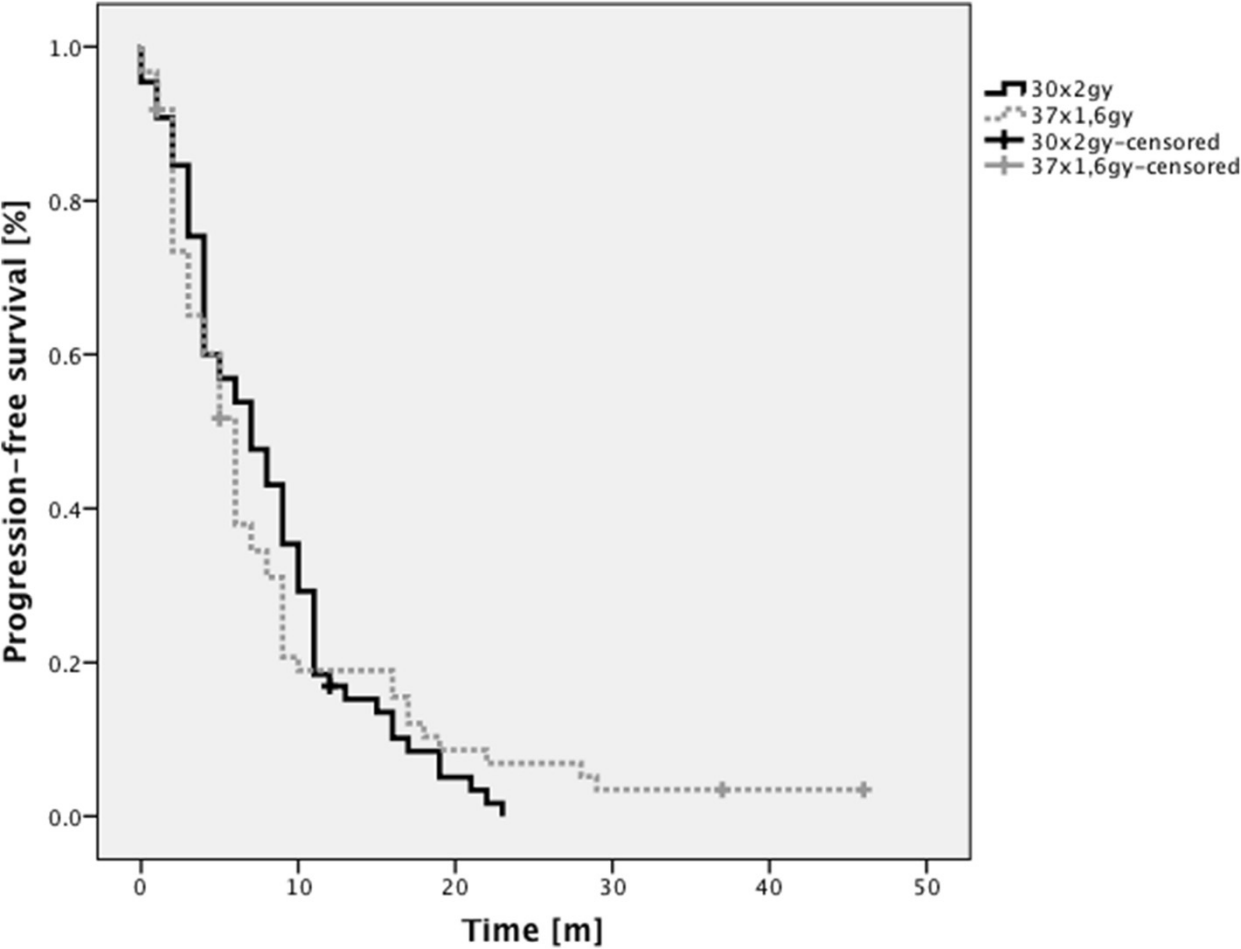
Kaplan-Meier analysis of PFS rates grouped according to dose regimen. No significant differences were found between both groups

Median PFS was 6 months for the entire cohort (Table 2). For patients treated with NFRT median PFS was 7 months, for patients treated with AHFRT median PFS was 6 months. At 6 months PFS was 56.9 % in the NFRT group and 51.7 % in the AHFRT group. At 12 months PFS was 16.9 % in the NFRT group and 19 % in the AHFRT group, (Fig. 1). There was no difference between both dose regimens in univariable analysis (*p* = 0.95).

**Table 2:** Univariable analysis of potential preditive factors of progression-free survival

	Univariable analysis				Multivariable analysis		
Variable	HR	95 % CI	*p*	Median PFS [m]	HR	95 % CI	*p*
Age (< vs. > = median of 61 years)	1.08	0.75–1.55	0.69	6 vs. 6	–	–	–
Gender (m vs. f)	0.68	0.46–1.01	0.05	6 vs. 9	0.57	0.35–0.92	0.022 (*)
KPS (< vs. > = median of 70 %)	0.5	0.34–0.72	<0.001 (*)	4 vs. 9	0.5	0.33–0.78	0.002 (*)
MGMT-status (methylated vs. unmethylated)	1.46	0.97–2.2	0.07	9 vs. 6	1.61	1.03–2.52	0.036 (*)
Localization (other vs. central)	1.51	0.76–3	0.24	6 vs. 5	–	–	–
PTV (< vs. > = median of 337 ccm)	1.13	0.79–1.62	0.51	7 vs. 6	–	–	–
Subtotal resection or biopsy vs. gross total resection	0.71	0.49–1.02	0.07	4 vs. 8	–	–	–
Fractionation regimen (NFRT vs. AHFRT)	1.01	0.95–1.01	0.95	7 vs. 6	–	–	–

(*) *p*-value ≤ 0.05, *HR* hazard ratio, *CI* confidence interval, *PFS* progression-free survival, *KPS* Karnofsky performance status, *MGMT* O-6-methylguanine-DNA methyltransferase, *PTV* planning target volume, *NFRT* normofractionated radiotherapy, *AHFRT* accelerated hyperfractionated radiotherapy

### Overall survival

Of 131 patients analyzed 107 had died at the time of analysis (01/2015).

**Fig. 2:**
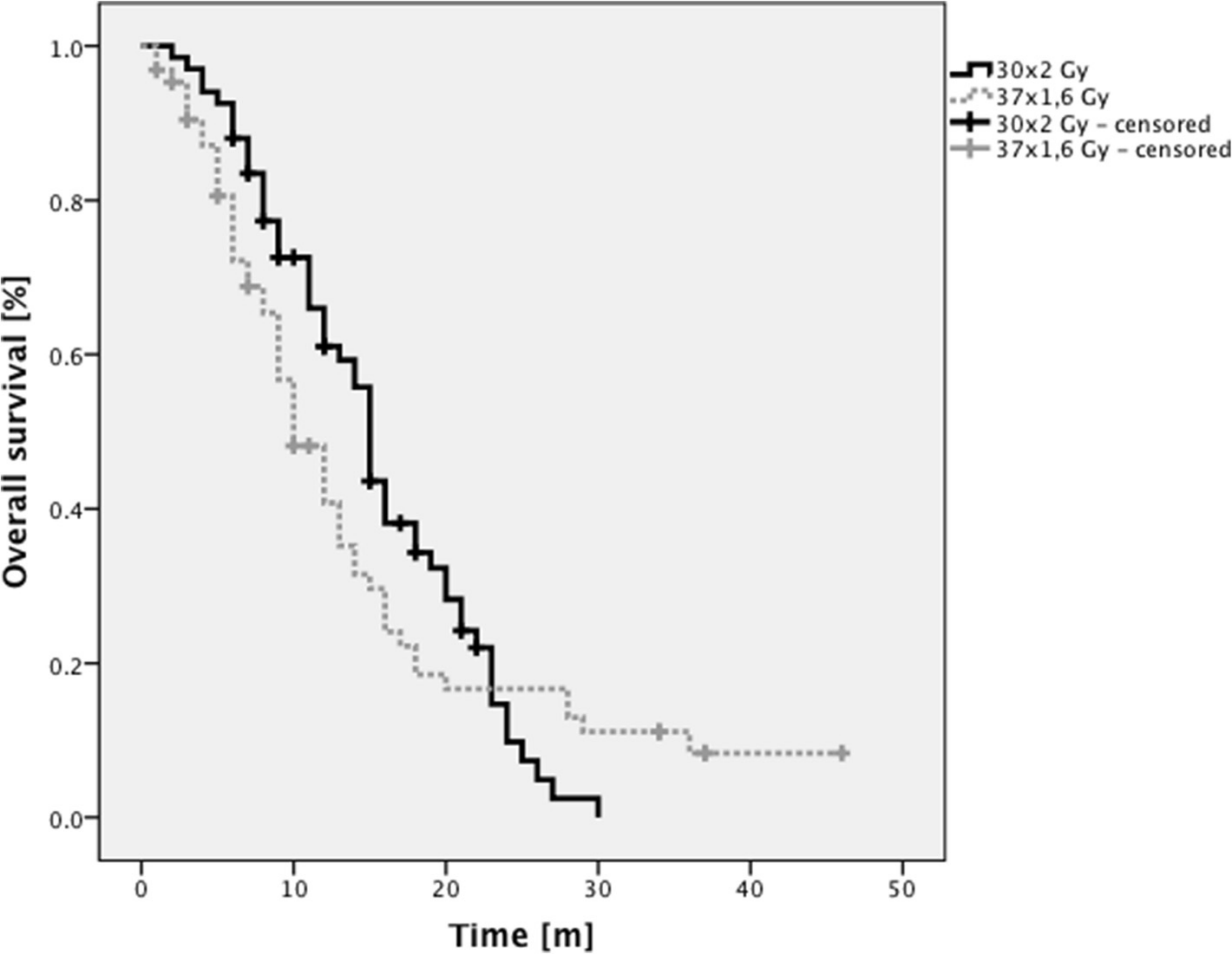
Kaplan-Meier analysis of OS rates grouped according to dose regimen. No significant differences were found between both groups

Median OS was 13 months for all patients (Table 3). For patients treated with NFRT median OS was 15 months, for patients treated with AHFRT median OS was 10 months. At 12 months OS was 66 % in the NFRT group and 48.2 % in the AHFRT group. At 24 months OS was 14.7 % in the NFRT group and 16.7 % in the AHFRT group (Fig. 2). There was no difference between both dose regimens in univariable analysis (*p* = 0.46).

**Table 3:** Univariable analysis of potential preditive factors of overall survival

	Univariable analysis			Multivariable analysis			
Variable	HR	95 % CI	*p*	Median OS [m]	HR	95 % CI	*p*
Age (< vs. > = median of 61 years)	1.18	0.8–1.7	0.4	14 vs. 12	–	–	–
Gender (m vs. f)	0.62	0.4–0.95	0.028 (*)	11 vs. 16	0.64	0.38–1.08	0.095
KPS (< vs. > = median of 70 %)	0.96	0.94–0.98	<0.001 (*)	9 vs. 15	–	–	–
MGMT-status (methylated vs. unmethylated)	1.68	1.08–2.61	0.021 (*)	16 vs. 11	1.89	1.158–3.09	0.011 (*)
Localization (other vs. central)	1.71	0.83–3.56	0.15	13 vs. 13	–	–	–
PTV (< vs. > = median of 337 ccm)	1.37	0.93–2.02	0.11	14 vs. 12	1.61	1–2.6	0.048 (*)
Subtotal resection or biopsy vs. gross total resection	0.64	0.43–0.95	0.025 (*)	11 vs. 15	0.62	0.39–0.98	0.041 (*)
Fractionation regimen (NFRT vs. AHFRT)	1.16	0.79–1.71	0.46	15 vs. 10	–	–	–

(*) *p*-value ≤ 0.05, *HR* hazard ratio, *CI* confidence interval, *OS* overall survival, *KPS* Karnofsky performance status, *MGMT* O-6-methylguanine-DNA methyltransferase, *PTV* planning target volume, *NFRT* normofractionated radiotherapy, *AHFRT* accelerated hyperfractionated radiotherapy

### Prognostic factors

Positive predictors of survival in univariable analysis were female gender, higher KPS, MGMT methylation and gross total resection. In multivariable analysis MGMT methylation and gross total resection remained significant predictors, the factor “smaller PTV” became significant in multivariable analysis. Gender and lower KPS were not significant in multivariable analysis.

The fractionation regimen was not a predictor of survival in univariable- or multivariable analysis.

Subgroup analysis according to predictive factors did not reveal any specific group to benefit from either NFRT compared to AHFRT or vice versa (Table 4).

**Table 4:** Subgroup analysis of potential preditive factors of overall survival did not identify any specific subgroup to benefit from either NFRT compared to AHFRT or vice versa

		Median OS [m]		
		NFRT	AHFRT	*p*
Variable				
Age	< median of 61 years	15	12	0.66
	> = median of 61 years	15	9	0.28
Gender	m	14	9	0.31
	f	16	14	0.98
KPS	< median of 70 %	12	6	0.16
	> = median of 70 %	15	13	0.67
MGMT-status	methylated	16	15	0.73
	unmethylated	14	9	0.09
Localization	other	15	10	0.41
	central	9	17	0.44
PTV	< median of 337 ccm	15	12	0.82
	> = median of 337 ccm	15	9	0.24
Extent of resection	Subtotal resection or biopsy	13	8	0.14
	gross total resection	15	13	0.6

*KPS* Karnofsky performance status, *MGMT* O-6-methylguanine-DNA methyltransferase, *PTV* planning target volume, *NFRT* normofractionated radiotherapy, *AHFRT* accelerated hyperfractionated radiotherapy

### Toxicity

All patients in both groups completed radiotherapy. All patients scheduled for concurrent chemotherapy (126/131) completed concurrent TMZ. In the normofractionated group seven patients did not complete post-radiotherapy TMZ due to neutropenia or thrombocytopenia. In the hyperfractionated group 3 patients did not complete post-radiotherapy TMZ due to neutropenia or thrombocytopenia.

There was no difference in acute toxicity profiles between the two treatment groups. There were seven grade 3 and six grade 4 events in the normofractionated group (grade 3 events: 1 × headache, 2 × neurological, 3 × neutropenia, 1 × thrombocytopenia. Grade 4 events: 2 × neutropenia and 4 × thrombocytopenia).

In the hyperfractionated group there were two grade 3 events and six grade 4 events (grade 3 events: 1 × neurological, 1 × nausea/vomiting. Grade 4 events: 3 × neutropenia, 3 × thrombocytopenia).

## Discussion

### Survival

Most studies on hyperfractionation and accelerated hyperfractionation stem from the pre-TMZ era, comparability of PFS and OS rates is thus limited. In our study median OS was 13 months for all patients, 15 months for patients treated using NFRT and 10 months for patients treated with AHFRT. Univariable and multivariable analysis did not show significant differences between the fractionation regimens. This is worthwile to know, because an AHFRT-regimen with 3.5 weeks overall treatment time was capable to equalize the OS-results of the classical 6 weeks treatment. Bearing in mind the limited prognosis of these patients the dose-intensified treatment is a clear benefit.

One of the first studies on AHFRT in GBM was published in 1994 by González et al. who used doses of 42–60 Gy in 2 Gy fractions three times a day. Median survival was 8.7 ± 0.7 months and no statistically significant differences were found for the four different dose-level groups [13].

Lutterbach et. al. published median OS rates of 8.8 months for 1.5 Gy thrice daily to 54 Gy [14].

In 2001 Prados et al. published survival rates of patients treated with AHFRT ± DFMO vs. conventional irradiation ± DFMO with no OS benefit for the experimental groups (8.6–9.8 months) [15].

Werner et al. published the RTOG 83–02 data in 1996, patients received HFRT (2 × 1.2 Gy to doses of 64.8, 72, 76.8, or 81.6 Gy) vs. AHFRT (2 ×1.6 Gy to doses of 48 or 54.4 Gy), all groups received concurrent BCNU. Contrary to the other aforementioned studies HFRT patients who had received higher doses of 76.8 and 81.6 Gy showed superior survival compared to the AHFRT groups. The authors found median OS rates between 10.8 and 12.7 months [16].

In 2005 Stupp et al. published data demonstrating a survival benefit for GBM patients that received concurrent Temozolomide with postoperative radiation, with median survival of 14.6 months for patients receiving concurrent therapy versus 12.1 months for patients who received only radiotherapy [7]. This treatment has since become the standard of care for primary GBM and is referred to as the “Stupp regimen” in everyday clinical routine.

OS rates for all patients of 13 months as shown here are comparable to the data published by Stupp et al. and we did not find significant differences in OS between AHFRT and NFRT in our patient collective.

### Limitations

Our study had several limitations. Firstly, the two groups analyzed were not perfectly matched in terms of age. Secondly, the MGMT-status is unknown in approximately 20 % of patients in both treatment groups. Thirdly, no analysis of chronic toxicity was performed due to the intrinsic uncertainties of retrospective analysis. Fourthly, the number of patients analyzed here in both groups might simply be too low to find significant differences in survival between the both regimens. Fifthly, patients with GBM in close proximity to the brainstem were more likely to receive AHFRT, potentially biasing OS rates.

## Conclusions

The role of AHFRT in the TMZ era remains unclear. The potential benefits are a reduction of tumor repopulation as well as reduced late toxicity. Other benefits are immanent; the regimen does significantly shorten hospitalization time in a patient collective with highly impaired life expectancy. We propose that the role of AHFRT + TMZ should be further examined in future prospective trials.
